# Catalytic DxD motif caged in Asx-turn and Met–aromatic interaction attenuates the pathogenic glycosylation of SseK2/NleB2 effectors

**DOI:** 10.1038/s41598-022-22803-y

**Published:** 2022-11-11

**Authors:** Eunhee Koh, Uijin Kim, Hyun-Soo Cho

**Affiliations:** Department of Systems Biology, College of Life Science and Biotechnology, Yonsei University, Seoul, 03722 Republic of Korea

**Keywords:** Biochemistry, Structural biology

## Abstract

Pathogenic bacteria encode virulent glycosyltransferases that conjugate various glycans onto host crucial proteins, which allows adhesion to mammalian cells and modulates host cellular processes for pathogenesis. *Escherichia coli* NleB1, *Citrobacter rodentium* NleB, and *Salmonella enterica* SseK1/3 type III effectors fatally glycosyltransfer *N*-acetyl glucosamine (GlcNAc) from UDP-GlcNAc to arginine residues of death domain-containing proteins that regulate host inflammation, intra-bacterial proteins, and themselves, whose post-translational modification disrupts host immune functions and prolongs bacterial viability inside host cells. However, unlike the similar NleB1/SseK1/SseK3, *E. coli* NleB2 and *S. enterica* SseK2 show deficient GlcNAcylation and neither intra-bacterial glycosylation nor auto-glycosylation. Here, as the major factor in SseK2/NleB2 deficiency, we focused on the catalytic Asp-x-Asp (DxD) motif conserved throughout all O-/N-glycosyltransferases to coordinate Mn^2+^. All DxD motifs in apo-glycosyltransferases form Type-I-turns for binding Mn^2+^, similar to the ligand-bound DxD motif, whereas TcnA/SseK2/NleB2 DxD motifs form Asx-turns, which are unable to bind Mn^2+^. Interestingly, methionine of the NleB2 DMD motif forms triple Met–aromatic interactions, as found in age-associated diseases and tumor necrosis factor (TNF) ligand-receptor complexes. The NleB1 A222M mutation induces triple Met–aromatic interactions to steeply attenuate glycosylation activity to 3% of that in the wild type. Thus, the characteristic conformation of the DxD motif is essential for binding Mn^2+^, donors, and glycosylate targets. This explains why SseK2/NleB2 effectors with the DxD motif caged in the Asp-/Asn-turn (Asx-turn) and triple Met–aromatic interactions have lower glycosyltransferase activity than that of other fatal NleB1/SseK1/SseK3 toxins.

## Introduction

Many bacteria produce protein toxins, which are released into the host cytoplasm to glycosylate targets and alter host defenses, such as immune-cell signaling, cytoskeletal organization, protein synthesis, and apoptosis^1,2^. *Clostridium difficile* causes antibiotic-associated diarrhea and pseudomembranous colitis, primarily mediated by two homologous exotoxins, *Clostridium difficile* toxins A (TcdA) and B (TcdB), which are prototypes of glycosylating toxins^3^. TcdA/B targets GTP-binding Rho proteins that act as molecular switches in regulating cell cycle progression by glucosylating the conserved threonine residues, which are essential for nucleotide binding and are located in the Rho switch-1 region to block Rho-dependent signaling^4^. Various clostridial species produce glycosyltransferase toxins related to TcdA/B^4^. *Clostridium sordellii* lethal toxin (TcsL) and *Clostridium novyi* α-toxin (TcnA) cause myonecrosis and toxic shock syndrome and induce soft tissue infections in injecting drug users by glycosylation at the same threonine residue of Rho proteins^5^. By comparing the TcdB sequence, a multidomain glucosyltransferase toxin (YGT) was found in the gram-negative bacterium *Yersinia mollaretii*. YGT N-acetylglucosaminylates (N-GlcNAcylates) are conserved threonine residues in the Ras superfamily Rab5/31, and inactivate the Rab switch in membrane trafficking^6^. Multidomain bacterial glycosyltransferase toxins, including prototypes TcdA/B, TcsL, TcnA, and YGT, are endocytosed to reach acidic endosomal compartments, and their pathogenic N-terminal glucosyltransferase domains translocate into the cytosol after cysteine protease auto-processing^7^ to catalyze O-linked glycosidic bond formation at canonical serine/threonine target residues using the sugar donors UDP-Glc/GlcNAc and cation cofactor^7^.

In addition to auto-processing translocation, many gram-negative bacterial pathogens use the bacterial type III secretion system (T3SS) of a needle-like pore spanning both their cell envelope and target host membrane to directly deliver their pathogenic effectors into host cells and inhibit cytokine release and inflammatory responses by the target cells^8,9^. Through the T3SS system, the enteropathogenic and enterohemorrhagic *Escherichia coli* (EPEC and EHEC), *Citrobacter rodentium* NleB effectors, and *Salmonella enterica* SseK1/2/3 effectors are released in the mammalian cell cytoplasm and covalently attach *N*-acetylglucosamine (GlcNAc) to the conserved arginine residues in several host targets such as cell death domain-containing proteins to block host cell apoptosis or necroptosis. Unlike the canonical O-linked glycosyltransferase toxins at the serine/threonine residues, NleB/SseK orthologs modify the uncommon guanidine group of a target arginine residue, which is known to be a poor nucleophile with partially delocalized electrons at physiological pH^10–14^.

A new glycosylation mechanism in arginine N-GlcNActransferase could be unpacked by solving the crystal structures of apo SseK2 and NleB2, UDP-bound SseK1 and SseK2, and the whole UDP-GlcNAc bound SseK2^2^ and NleB1 in the free ligand state as well as in complex with both UDP and acceptor Fas-associated death domain (FADD)^15^, and apo and UDP-bound SseK3 structures^16,17^. All NleB/SseK orthologs have a typical GT-A conformation with two abutting β/α/β Rossmann domains unlike a GT-B consisting of facing each other β/α/β Rossmann domains with a flexible linkage and share a high degree of structural similarity^2,15–17^. Several structural cores were revealed for the new arginine N-glycosylation systems: a catalytic DxD motif to position the Mn^2+^ cofactor and UDP-GlcNAc donor substrate, His-Glu-Asn (HEN) motif to select UDP-GlcNAc as a suitable donor substrate before other sugar donors such as UDP-Glc/Gal, C-terminal loop to stably embrace the UDP-GlcNAc donor substrate, and Helix-Loop-Helix (HLH) domain predicted to select the acceptor targets^2,15–17^.

NleB/SseKs orthologs GlcNAcylate several host death domains involved in regulating apoptosis and inflammation acceptors, such as Fas-associated death domain (FADD), tumor necrosis factor receptor type 1-associated death domain protein (TRADD), receptor-interacting serine/threonine-protein kinase 1 (RIPK1)^11,12^, the death domains in receptors of the mammalian tumor necrosis factor (TNF) superfamily TNFR1 and TRAILR^17^, as well as host glyceraldehyde 3-phosphate dehydrogenase (GAPDH)^18^, ensconsin (MAP7)^19^, hypoxia-inducible factor 1-alpha (HIF1a)^20^, and Rab small GTPases^21^, which subvert their antibacterial functions and inhibit inflammatory host responses. Moreover, before being released into host cells, NleB/SseKs orthologs N-GlcNAcylate the arginine residues of their intra-bacterial enzymes inside the bacterial cytosol. *C. rodentium* NleB and *E. Coli* NleB1 toxins GlcNAcylate glutathione synthetase (GshB) to enhance GshB activity and survival under oxidative stress conditions^22^. The SseK1 effector GlcNAcylates Arg9, Arg190, Arg160, and Arg149 of four proteins involved in methylglyoxal detoxification, namely, GloA, GloB, GloC, and YajL, respectively, and catalyze methylglyoxal detoxification to significantly enhance their activity^13^. In addition to a variety of host or intra-bacterial target proteins, the NleB/SseK ortholog itself is also auto-GlcNAcylated by attaching N-acetylglucosamine to several arginine guanidine groups at Arg13/53/159/293 in NleB1, Arg30/158/339 in SseK1, and Arg153/184/305/335 in SseK3^2,17,23^.

Here, *C. rodentium* has only one fatally pathogenic NleB effector, whereas EPEC and EHEC possess two distinct NleB orthologs: the NleB1/2 variants. NleB1 is fatally pathogenic with high GlcNAcylation, intra-bacterial glycosylation, and auto-glycosylation, whereas the NleB2 effector shows no signal in the GlcNAcylation of several NleB1 host targets, intra-bacterial glycosylation, or auto-glycosylation. Similarly, *S. enterica* produces three homologous SseK1/2/3 effectors, of which the SseK2 effector shows weaker GlcNAcylation activity than that of the fatal SseK1/3 toxins and has no signal in intra-bacterial glycosylation or auto-glycosylation, unlike the SseK1/3 effectors.

However, it is unclear why SseK2/NleB2 effectors show a weaker glycosyltransferase signal despite more than 60% sequence identity and less than 0.90 Å RMS structure similarity with the fatal SseK1/2 and NleB1 effectors^14^. As a major catalytic factor for deficient SseK2/NleB2 glycosylation, we focused on the catalytic Asp-x-Asp (DxD) motif, which is essential for binding Mn^2+^ and UDP-GlcNAc, which is conserved throughout all mono-glycosyltransferase bacterial toxins regardless of the release mechanism and O-/N-glycosylation.

First, we resolved the hydrogen-bond structure in the DxD motif of the apo NleB2 effector by orienting one water molecule adjacent to the DxD motif and processing repeated refinements of the NleB2 crystal structure. All solved N-glycosyltransferase NleB/SseKs structures were revisited to analyze the characteristic topology of the catalytic DxD motif and this analysis was extended to all structures of O-linked bacterial glycosylation toxins to determine whether these DxD motifs follow the characteristic topology. This revealed that in all ligand-bound N-/O-glycosylation toxins, the backbone of the DxD motif forms a Type I β-turn, in which the four residues (labeled i to i + 3) specifically form a pseudo-cycle C10 H-bond network between the backbone CO(i) and NH(i + 3) groups, and that the first aspartate binds with its next-but-one tyrosine, named as the YD bond, which allows the DxD motif to optimally bind Mn^2+^ and the donor substrates. In all O-glycosyltransferase toxins, except for one apo TcnA, the DxD motif retains a Type-I-turn with the first aspartate in the YD-bond and is ready to bind Mn^2+^. In particular, the DxD motif of apo TcdA/B toxins can bind Mn^2+^ without binding ligands. However, in SseK2/NleB2 effectors, we found that the first aspartate in the DxD motif was previously intertwined with the C10 H-bond network, namely the Asx-turn (Asp/Asn-turn), which is incapable of binding Mn^2+^. Recently, Asn-containing peptide models, specifically designed to not form the β-turn, favor intrinsic folding into an Asx-turn; analysis of Asn turns in PDB proteins confirmed that the Asx-turn is the most stable innate structure before the backbone β-turn^24^. This suggests that the catalytic aspartate side-chain of the SseK2/NleB2 DxD motif caged-in Asx-turn is more tightly intertwined than the free aspartate side-chain of the fatal SseK1/3 and NleB1 DxD motifs in the backbone Type-I-turn, which prohibits the catalytic DxD motif from binding Mn^2+^ and glycosylating targets.

Furthermore, unlike alanine in other pathogenic NleB/SseKs DxD motifs (DAD), the deficient NleB2 has rigid methionine between two catalytic aspartates of the NleB2 DxD motif (DMD). In the newly refined NleB2 crystal structure, we found that the single methionine in the NleB2 DMD motif was located at the center of three aromatic residues (Phe, Tyr, and Trp) within a distance of 6.2 Å to form triple Met–aromatic interactions with the three neighboring aromatic rings at an angle of approximately 55°. Met–aromatic binding between the sulfur in methionine and aromatic rings of Phe, Tyr, and Trp residues is prevalent in known protein structures and provides additional stabilization of 1–1.5 kcal/mol over purely hydrophobic interactions^25,26^; further, it is associated with several age-associated diseases, including Alzheimer’s, Creutzfield–Jacob, and von Willebrand diseases. In von Willebrand disease, a single valine replacing Met within a 5 Å distance from three aromatic residues results in an overactive glycoprotein Ibα, which is associated with a bleeding disorder^27,28^. The wild-type prion protein has a methionine with 5.5 Å separation from three aromatic tyrosine residues^29^ and the mutation of prion protein at Met129 is an allelic risk factor for the development of neurodegenerative diseases, such as Alzheimer^30^ as well as fatal familial insomnia and familial Creutzfield–Jacob^31^. In addition to age-associated diseases, NleB/SseKs-relevant TNF ligand-receptor binding in the TNF-related apoptosis-inducing ligand in complex with death receptor 5 (TRAIL-DR5) and lymphotoxin-ligand in complex with TNF receptor 1 (LT-TNFR1) are critically stabilized by Met–aromatic interactions, where a single replacement of DR5 Met99 dramatically reduced ligand-binding to 18% compared to that with the wild type^32^. Here, we suggest that the triple Met–aromatic interactions of the NleB2 DMD motif inhibit the free binding of the two catalytic aspartates with Mn^2+^. To confirm the negative effect of the DMD motif on glycosylation, we constructed an NleB1 A222M variant from the pathogenic NleB1 DAD motif, in which the mutated Met is placed at the center of three aromatic residues (Phe, Tyr, and Trp) that form three Met–aromatic bonds, and the NleB2 M219A variant from the NleB2 DMD motif to remove the Met–aromatic bond. We further performed UDP-Glo assays for NleB1 A222M to show that the kinetic parameters Vmax and Km in NleB1 A222M were dramatically reduced compared to those of the wild-type NleB1 and NleB2 M219A variants, whose Km was increased compared to that of the wild-type NleB2.

Taken together, we suggest that the characteristic conformation of the DxD motif is essential for the binding of Mn^2+^ with the donor and to further glycosylate its targets. This can explain why SseK2/NleB2 effectors with the DxD motif caged in Asx-turn and Met–aromatic bonds have low glycosyltransferase activity compared to that of the other fatal NleB1/SseK1/SseK3 toxins.

## Results

### Refinement of the Apo NleB2 structure

**Figure 1:**
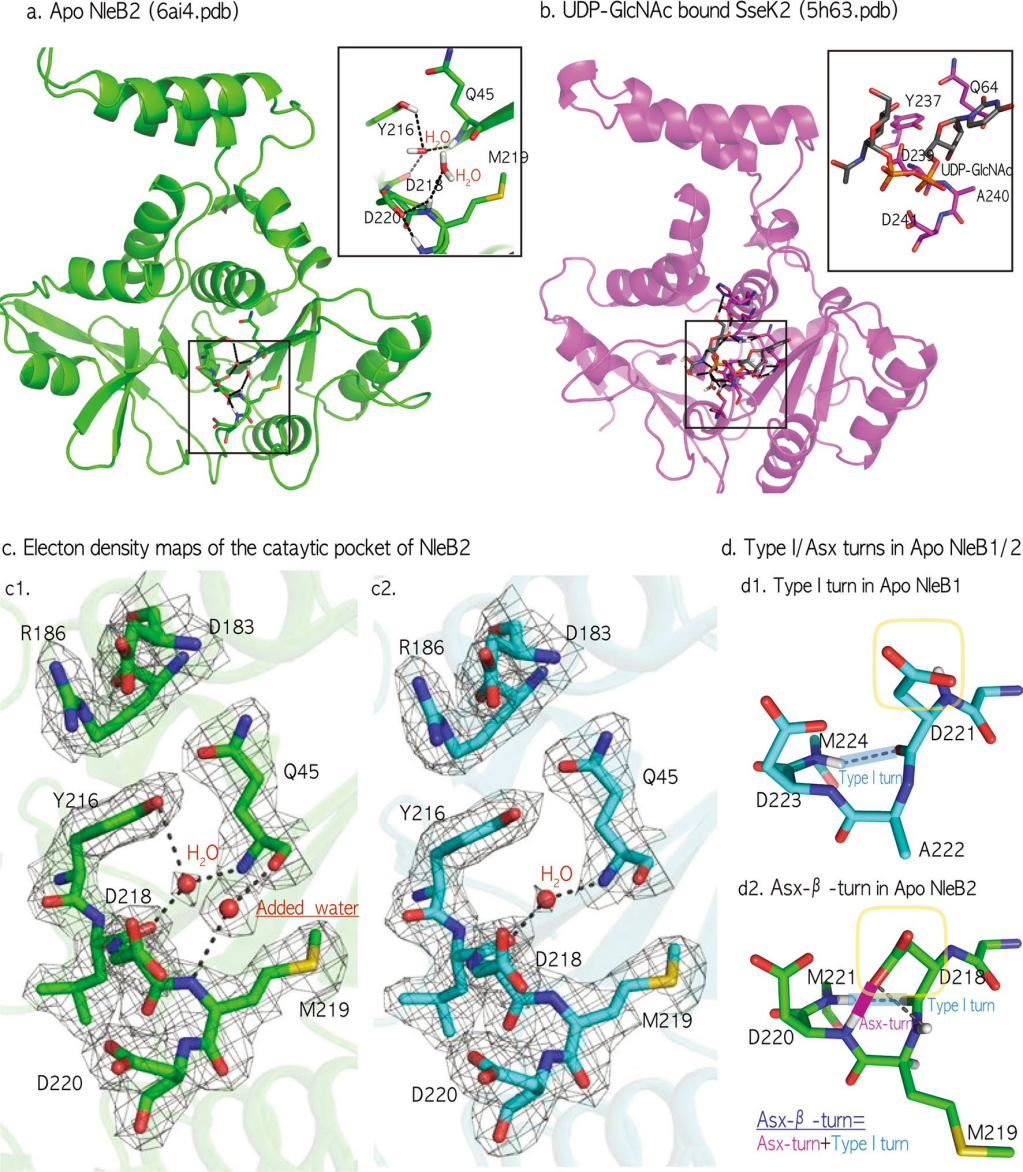
Refinement of the apo NleB2 structure. (**a**) Apo NleB2 Refinement of apo NleB2 (1–316) resulted in better resolution of 2.10 Å from the old 2.30 Å (5H5Y) and showed the detailed network between catalytic cores Q45, Y216, D218, and D220 including three residues Asp218–Met219–Asp220 of DxD motif, comparing with the catalytic pocket of the whole SseK2 in the UDP-GlcNAc bound complex (**b**). (**c**) Electron density maps for a newly oriented water in the catalytic pocket of NleB2. Electron density maps for the DxD motifs of NleB2 and their interacting water molecules of 6ai4 (**c1**) and 5h5y (**c2**) 2Fo-Fc electron density maps are contoured at the 1.1σ level (colored gray). Hydrogen bonds are shown as black dashed lines, Q45, Y216, and DMD motifs are represented as sticks and water molecules are depicted as red balls. In the refinement process, we added one water molecule to clarify the connection network among Tyr216, Gln45, and triple residues Asp218-Met219-Asp220 of the DxD motif and confirmed that the new water concretely connects the amino group NH of Met219, the carboxyl group CO of Gln45, the hydroxyl oxygen atom Oη of Tyr216 and one of the carboxylate oxygen atoms Oδ of Asp218 in the electron density map, unlike the previous apo NleB2 (5H5Y). (**d**) The Asx-turn in Apo NleB2 *vs*. the Type-I-turn in NleB1. The Apo NleB1 DxD motif adopts a Type-I-turn defined by four consecutive residues (221DADM224) and an H-bond between the CO of D221 and NH of M224 (**d1**) The refined apo NleB2 DxD motif showed that the side chain of the first aspartate Asp 218 in DxD motif makes Asx-turns, combined with another secondary structure Type-I-turn between the CO of D218 and NH of M221, which form an intertwined C10 H-bond network, labeled as an Asx-β-turn (**d2**).

To clarify the hydrogen-bond topology of the apo NleB2 (1–316) DxD motif, one water molecule was newly oriented adjacent to the DxD motif and several refinements were processed by molecular replacement with the UDP-GlcNAc bound SseK2 structure. Repeated refinement of apo NleB2 improved the resolution to 2.10 Å from the old 2.30 Å of 5H5Y and resulted in the detailed hydrogen-bond structure in a catalytic pocket including three residues Asp218-Met219-Asp220 of the DxD motif (Fig. 1a,b). In particular, one added water molecule manifested an H-bond network among Tyr216, Gln45, and the triple residues Asp218-Met219-Asp220 of the DxD motif, in which the water molecule strongly connected the amino group NH of Met219, the carboxyl group CO of Gln45, the hydroxyl oxygen atom Oη of Tyr216, and carboxylate oxygen atom Oδ of Asp218 in the electron density map; this was unsolved in the previous NleB2 structure (Fig. 1c).

In the apo NleB1 DxD motif (221**DAD**M224), the four residues (labeled i to i + 3) specifically form a pseudo-cycle C10 H-bond network between the backbone CO(i) and NH(i + 3) groups, referred as a Type I β-turn, and the Apo NleB2 DxD motif (218**DMD**M220) also conforms to the backbone Type-I-turn (Fig. 1d). Unlike the NleB1 toxin (Fig. 1d), apo NleB2 216YL**DMD**M221 has an extra H-bonding pattern, namely an Asx-turn, where the backbone NH(k + 3) group hydrogen-bonds with the side chain of the Asn/Asp(k + 1) residue, and not with the backbone CO(k) in the four residues (labeled k to k + 3). In detail, Oδ of the D218 sidechain in apo NleB2 216YL**DMD**M221 interacts with the next-but-one NH of D220 along the backbone, giving rise to an Asx-turn combined with another Type-I-turn that forms a characteristically twice-intertwined C10 H-bond network, labeled as an Asx-β-turn (Fig. 1d).

**Figure 2:**
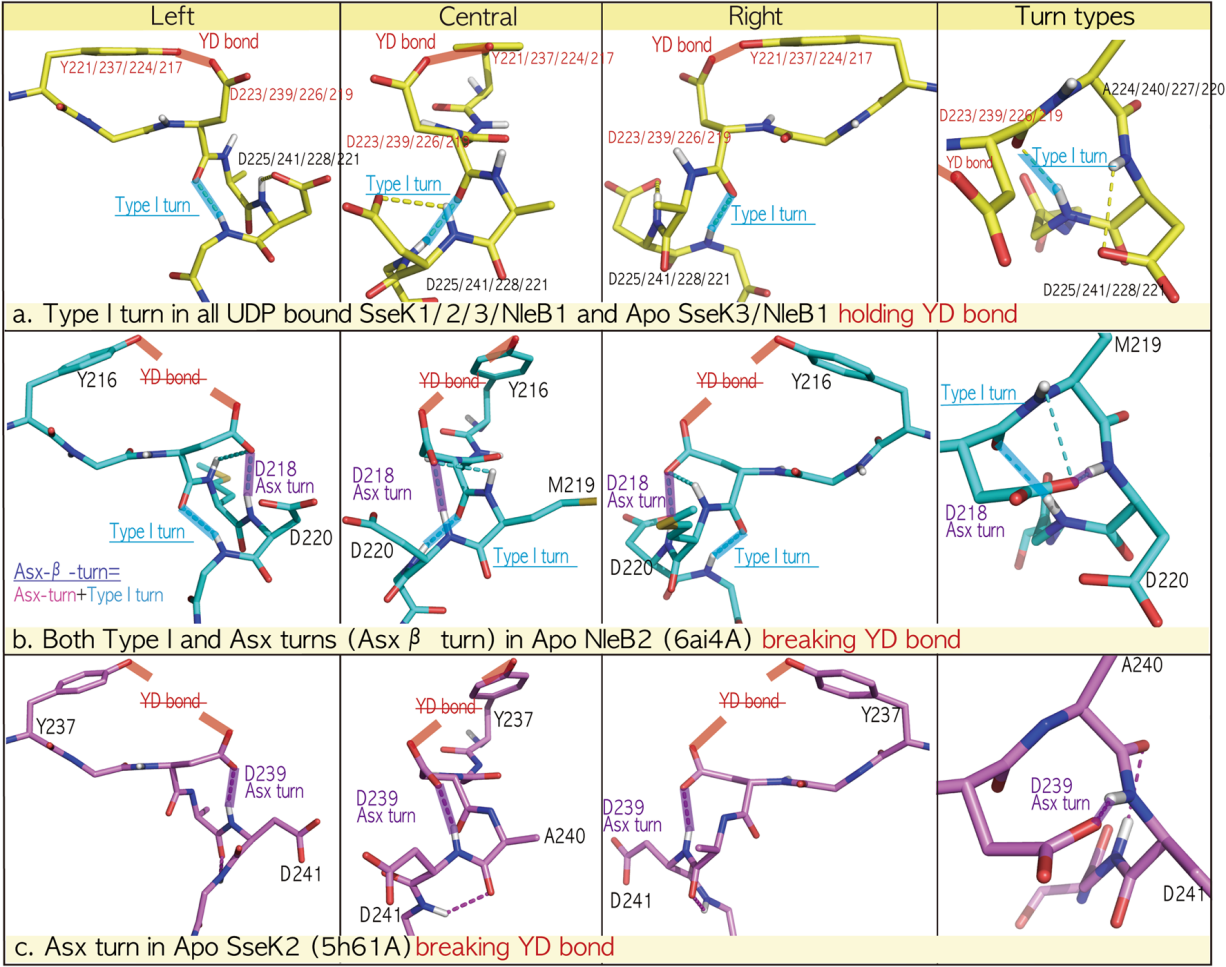
Type-I-turns in fatal NleB1/SseK1/SseK3 *vs*. the Asx-turn in SseK2/NleB2. (**a**) Type-I-turns in all ligand-bound SseK1/2/3/NleB1 and apo SseK3/NleB1 DxD motifs holding the YD bond. In the ligand-bound complex of all NleB/SseKs orthologs and apo NleB1/SseK1/SseK3 effectors (Table 2), the DxD motif folds to a Type-I-turn (cyan colored), and the sidechain of tyrosine holds the first aspartate side chain, referred to as a YD bond (red colored). (**b**) Both Type I and Asx-turns (Asx β turn) in apo NleB2 (6AI4) break the YD bond. The refined NleB2 DxD motif showed the characteristic Asx β turn, twice intertwined with Type-I-turn (cyan colored) and Asx-turn (purple colored) by two C10 H-bond networks between backbone CO of D218 and NH of M221 and between the side chain of D218 and NH of D220, resulting in breaking the YD bond. (**c**) Asx-turn in apo SseK2 (5H61: < Chain A) breaks the YD bond. In apo SseK2, the DxD motif forms an Asx-turn, by a C10 H-bond network between the side chain of D239 and the backbone NH of D241, resulting in breaking the YD bond.

In all apo- and UDP-bound NleB1/SseK1/SseK3 effectors, the DxD motifs lie in the 219YL**DAD**M224/221YL**DAD**M226/224YL**DADM**229 regions and their quaternary DADM motifs form a Type-I-turn by H-bonding between the backbone CO(i) and NH(i + 3) groups, while the free Asp oxygen (Oδ of Asp221/Asp223/Asp/226) in the DxD motif interacts with the hydroxyl oxygen (Oη of Tyr219/Tyr221/Tyr224), named as a YD bond (Fig. 2a). In particular, apo NleB1/SseK1/SseK3 effectors have the free oxygen Oδ of the first catalytic aspartate in the DxD motif, ready to catch Mn^2+^ and UDP-GlcNAc (Fig. 2a).

Surprisingly, the alternative Asx-turn of apo NleB2 is also found in the apo crystal structure of another *S. enterica* SseK ortholog, SseK2. In the SseK2 237YL**DAD**M242 region, the carboxylate oxygen Oδ of D239 interacts with the backbone NH group of D241, forming the same pseudo-cycle as the Asx-turn (Fig. 2b,c). In both apo NleB2/SseK2, the catalytic aspartate oxygen Oδ of Asp218/Asp239 is tightly intertwined in the Asx-turn and is unable to bind Mn^2+^, which is different from the free oxygen Oδ of the DxD motif in the other pathogenic NleB1/SseK1/SseK3 effectors (Fig. 2). Although apo SseK2 has only one pseudo-cycle Asx-turn, the apo NleB2 DxD motif has twice the intertwined Asx-β-turns as one Asx-turn and another Type-I-turn, because of which it is more tightly tied than SseK2 DxD (Fig. 2b,c).

### Conserved type-I-turn in the DxD motif throughout all fatal bacterial O-/N-linked glycosylation toxins

**Figure 3:**
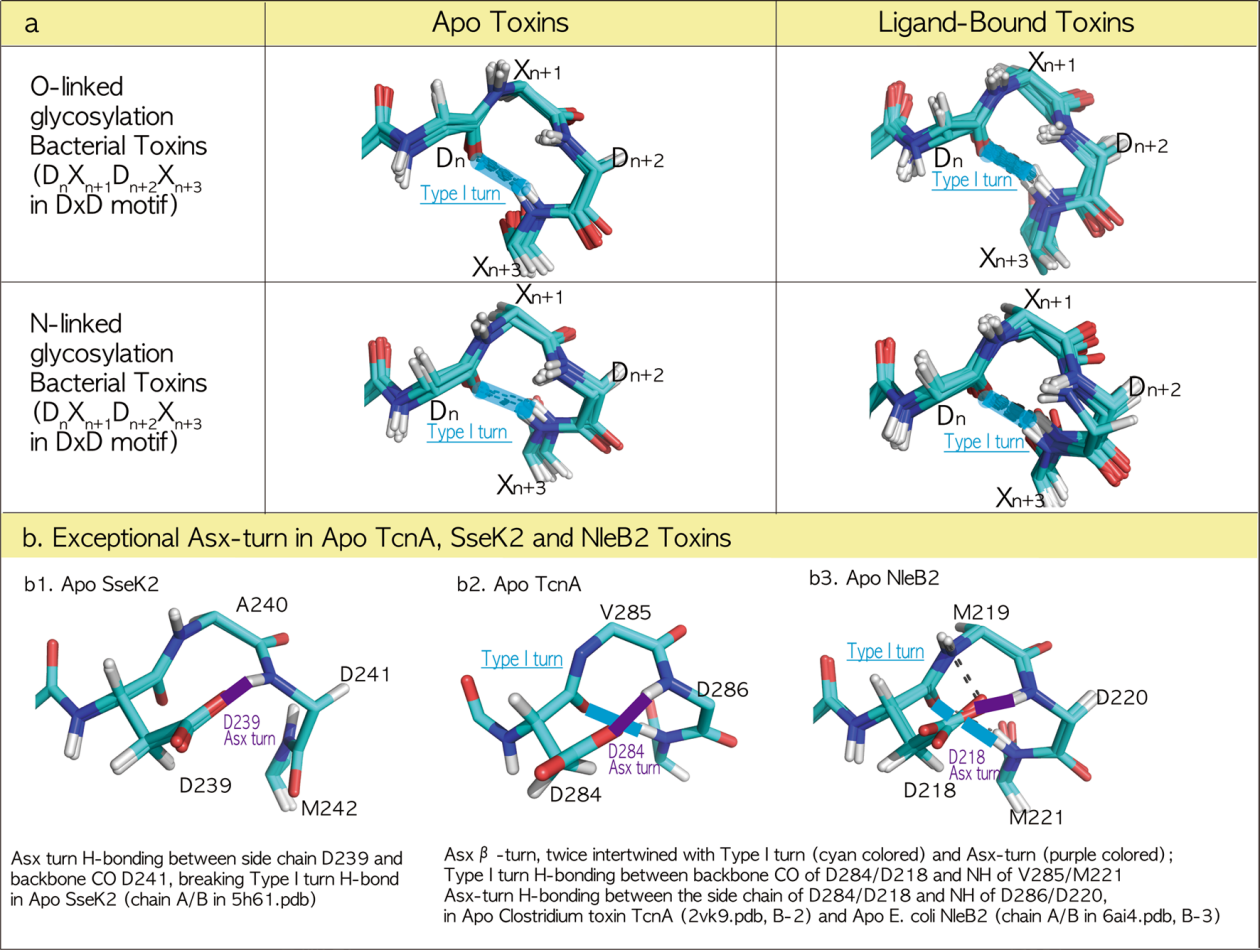
Conserved Type-I-turn in all fatal bacterial glycosylation toxins, regardless of O-/N-linkage. (**a**) Type-I-turn in the DxD motif of all glycosylation toxins regardless of the binding ligand and O-/N-linked glycosylation. All 30 solved crystal structures of glycosyltransferase toxins were gathered from the RCSB PDB site and a total of 51 chains in the asymmetric unit were separated into four groups consisting of apo (11 chains) and ligand-bound O-linked toxins (18 chains) and apo (7 chains) and ligand-bound N-linked NleB/SseKs toxins (15 chains) in Table 2. For apo (11 chains) and ligand-bound O-linked toxins (18 chains), the ten-residue region near the DxD motif in each chain (TcdA: 281GVYL**DVD**MLP290/TcdB: 282GMYL**DVD**MLP291/TcsL: 282GMYL**DVD**MLP291/TcnA: 280GVYC**DLD**FLP289/YGT: 207GVYIDTDLLP216) is structurally aligned with 281GVYL**DVD**MLP290 of TcdA (3SS1) and 281GVYL**DVD**MLP290 TcdA (3SRZ), respectively. For apo (7 chains) and ligand-bound N-linked NleB/SseKs toxins (15 chains), the ten-residue region near the DxD motif in each chain (SseK1: 219CIYLDADMII228/SseK2: 235CIYLDADMll244/SseK3: 222CIYLDADMll231/NleB1: 217CIYLDADMII226/NleB2: 214CIYLDMDMIL223) is structurally aligned with 222CIYLDADMll231 SseK3 6eyrA and 235CIYLDADMll244 SseK2 (5H63, B chain), respectively. In all bacterial mono-glycosylation toxins, the DxD motif folds to a Type-I-turn (cyan colored) regardless of the binding ligand or O-/N-linkage glycosylation, except for Apo SseK2 (Chain A/B in 5H61, **b1**), apo *Clostridium* toxin TcnA (2VK9, **b2**) and apo *E. coli* NleB2 (Chain A/B 6AI4, **b3**). (**b**) Exceptional Asx-turn in Apo TcnA, SseK2, and NleB2 toxins. The DxD motif in Apo SseK2 (Chain A/B in 5H61) forms an Asx-turn (purple colored) by C10 H-bonding between side chain D239 and backbone CO D241, breaking the Type-I-turn H-bond (**b1**). However, the apo *Clostridium* toxin TcnA (2VK9, **b2**) and apo *E. coli* NleB2 (Chain A/B in 6AI4, **b3**) have the specific C10 H-bond network in the DxD motif, called as Asx β-turn, twice intertwined with Type-I-turn (cyan colored) and Asx-turn (purple colored) by two C10 H-bond networks with Type-I-turn between backbone CO of D284/D218 and NH of V285/M221 and Asx-turn between the side chain of D284/D218 and NH of D286/D220, in apo TcnA (2VK9, **b2**) and apo NleB2 (Chain A/B in 6AI4, **b3**).

**Figure 4:**
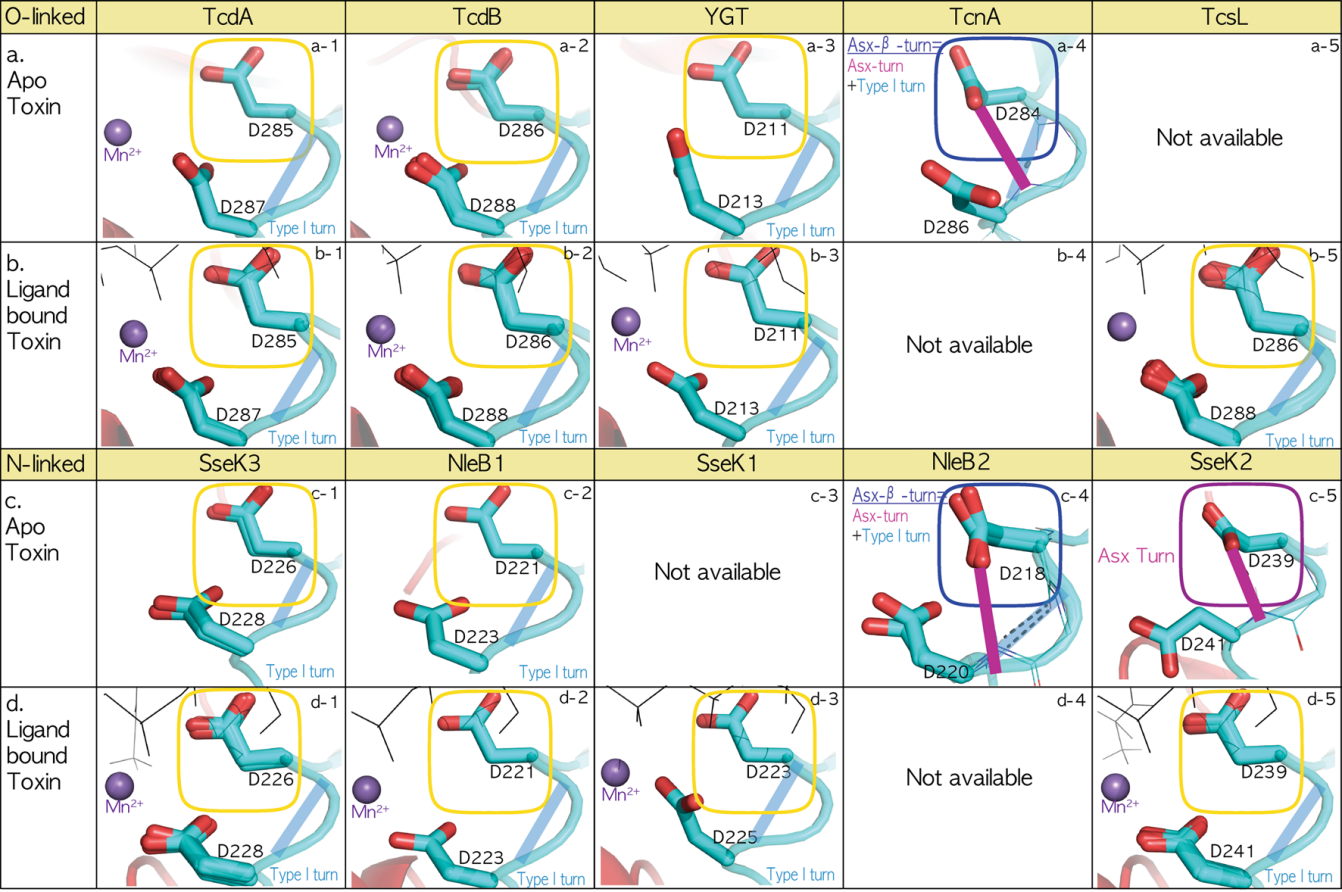
Pincher-like Aspartate Dn in the DxD (Dn-Xn + 1-Dn + 2) motif of bacterial mono-glycosylation toxins. All 51 known structures of O-glycosylation TcdA/B, TcsL, TcnA, and YGT, the N-glycosyltransferase NleB1/2, and SseK1/2/3 toxins in the asymmetric unit (Table 2) are separated into each toxin-related ligand-binding from apoO-glycosylation in row (**a**), ligand-bound O-glycosylation in row (**b**), apo N-glycosylation in row (**c**) and ligand-bound N-glycosylation in row d. All ligand-bound toxins have a Type-I-turn and a backbone C10 H-bond network, for the first aspartate side-chain in the DxD motif to freely bind the cofactor Mn^2+^ in rows (**b**) and (**d**). Except for the three toxins in apo TcnA, SseK2, and the NleB2 toxins (**a4**, **c4/5**), all glycosylation toxins retain the Type-I-turn in the DxD motif regardless of O-/N-linked glycosylation (cyan colored). The type-I-turn in the apo TcdA/B DxD motif as the backbone C10 H-bond network permits the first catalytic aspartate to coordinate with Mn^2+^ (3SS1, **a1** and 5UQT, **a2**). Unlike the first aspartate sidechain of the Apo SseK2 DxD motif in only the Asx-turn (5H61, **c5**), both TcnA (2VK9, **a4**) and NleB2 (6AI4, **c4**) DxD motifs are tightly tied by double C10 H-bond networks, an Asx-turn, twice intertwined with a Type-I-turn (cyan colored), and an Asx-turn (purple color).

All N-glycosylated NleB/SseKs form a Type-I-turn in the DxD motif in the ligand-bound state, and the pathogenic NleB1/SseK1/3 toxins also retain Type-I-turn in the DxD motif in the apo state (Fig. 2a). Next, we investigated whether other O-glycosylation toxins have this pattern of N-glycosylation. All 30 crystal structures of glycosyltransferase toxins were gathered from the RCSB PDB site, and a total of 51 chains in the asymmetric unit were separated into four groups comprising apo (11 chains) and ligand-bound O-glycosylation toxins (18 chains) and apo (7 chains), and ligand-bound N-glycosylation NleB/SseK toxins (15 chains) in Table 2. For the apoO-glycosylation toxin group, each ten-residue region near the DxD motif in 11 chains (Table 2) was structurally aligned to 281GVYL**DVD**MLP290 of TcdA (3SS1). For the ligand-bound O-glycosylation toxins, the ten-residue region in 18 chains (Table 2) was fitted to 281GVYL**DVD**MLP290 of TcdA (3SRZ). For apo (7 chains) and ligand-bound N-linked NleB/SseKs (15 chains), each ten-residue region (Table 2) was structurally aligned to 222CIYL**DAD**Mll231 SseK3 (6EYR) and 235CIYL**DAD**Mll244 SseK2 (5H63), respectively (Fig. 3a). Except for the three structures of apo TcnA, SseK2, and NleB2 toxins, all glycosylation toxins retained a Type-I-turn in the DxD motif regardless of the apo/ligand-binding and O-/N-glycosylation (cyan-colored, Fig. 3a). DxD motifs in the exceptional apo TcnA, SseK2, and NleB2 fold to an Asx-turn, where the first catalytic aspartate side chain is tightly tied to the second aspartate backbone (purple colored, Fig. 3a). In detail, all DxD motifs in apo SseK2 form an Asx-turn (purple colored) by C10 H-bonding between side chain D239 and backbone CO D241, breaking the Type-I-turn H-bond (Figs. 2c and 3a). Apo *Clostridium* toxin TcnA and apo *E. coli* NleB2 have twice intertwined Asx-β-turns, where Type-I-turns H-bond between the backbone CO of D284/D218 and the backbone NH of F287/M221, and the Asx-turn C10 H-bonds between the side chain of D284/D218 and NH of D286/D220 in apo TcnA/NleB2 (Fig. 3a). These results indicate that the first catalytic aspartate in Apo TcdA/B, TcsL, SseK1/3, and NleB1 DxD motifs is open to bind Mn^2+^, whereas those of Apo TcnA, SseK2, and NleB2 are tightly tied by the second aspartate backbone in the Asx-turn of the DxD motif and are unable to bind Mn^2+^ and their ligand. This difference in Mn^2+^ coordination between the Type-I-turn (TcdA/B, TcsL, SseK1/3, and NleB1) and Asx-turn (TcnA, SseK2, and NleB2) was confirmed by the existence of Mn^2+^ bound apo TcdA/B without a binding ligand, where the Type-I-turn in the TcdA/B DxD motif permits catalytic aspartate to easily coordinate with Mn^2+^ unlike the aspartate of Apo SseK2/TcnA/NleB2 DxD, which is tightly tied by an Asx-turn (Fig. 4a). Here, apo TcnA and NleB2 DxD motifs with the twice-intertwined Asx-β-turn could be more difficult to bind with Mn^2+^ as well as unable to hold two oxygens Oα-Pα and Oβ-Pβ of UDP-GlcNAc than those of SseK2 and other pathogenic TcdA/B, TcsL, SseK1/3, and NleB1 toxins (Fig. 4). This could explain why, like TcnA toxin^5^, several soaking experiments of NleB2 with UDP-GlcNAc and Mn^2+^ did not result in bound ligands despite free space in the active center packing, resulting in failed co-crystallization attempts, whereas SseK2 crystals with UDP-GlcNAc and Mn^2+^ could be obtained by soaking experiments^2^.

### Met–aromatic interaction in the NleB2 DMD motif is unlike that of DAD in the other NleB1/SseKs orthologs

**Figure 5:**
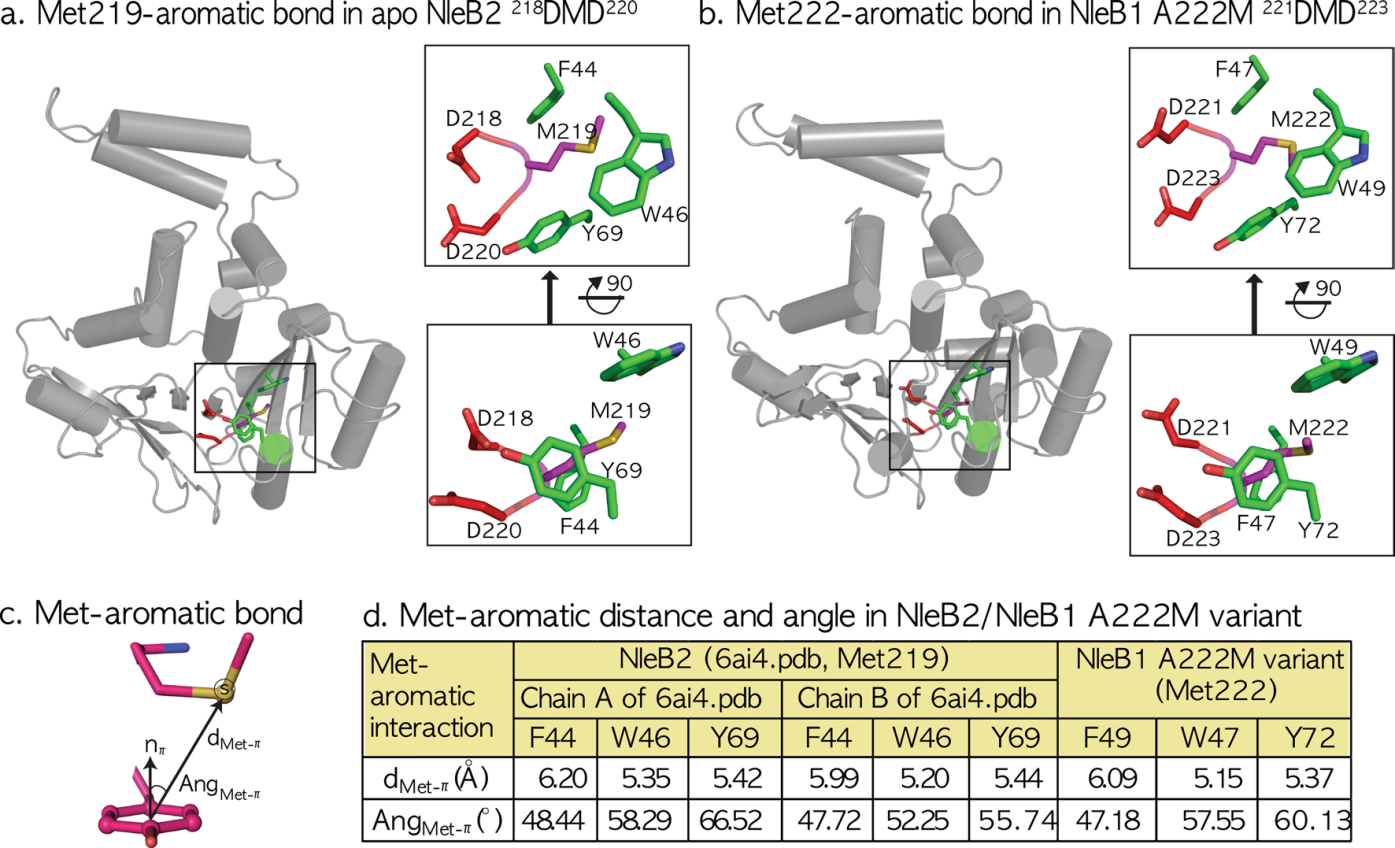
Met–aromatic interaction in NleB1/2 toxins and kinetic analysis of NleB1, NleB2, and their derivatives with FADD DD in the presence of UDP-Glc/GlcNAc. (**a**) Met219-aromatic interaction in apo NleB2. In the refined apo NleB2 structure, there are three aromatic residues, F44, W46, and Y69, near the methionine in the DxD (218DMD220) motif. (**b**) Met222-aromatic interaction in the NleB1 A222M variant. Substitution of Ala222 with Met222 in the wild NleB1 structure (6ACI) facilitates Met–aromatic interaction with the three aromatic residues, F47, W49, and Y72. (**c**) Met–aromatic bond. The Met–aromatic distance d_Met–π_ is measured by the distance between the sulfur of the Met residue and the ring center of three aromatic residues, Phe, Tyr, and Trp, and the orientation angle, Ang_Met–π_ is defined as the angle between the positional vector of sulfur from the ring center and the normal vector defined by the plane of the aromatic ring, as described in the “Methods”. (**d**) Met–aromatic distance and angle in NleB2/NleB1 A222M variant. All Met–aromatic interactions near the DxD motif were extracted as defined previously^32^, wherein the Met–aromatic energy is associated with both the distance d_Met–π_ and the orientation angle, Ang_Met–π_ between Met and three aromatic residues, Phe, Tyr, and Trp.

Among the NleB/SseKs orthologs, only NleB2 toxin has a distinct methionine between two aspartates in the DxD motif (218D**M**D220). All other orthologs, including SseK1 223D**A**D225, SseK2 239D**A**D241, SseK3 226D**A**D228, and NleB1 221D**A**D223 have an alanine at this position. Because of its greater hydrophobicity, the changed methionine in NleB2 D**M**D could hinder shifting for ligand binding than that with alanine in NleB1 and SseK1/2/3 D**A**D. Moreover, there are three aromatic residues, F44, W46, and Y69, near methionine in NleB2 DMD, resulting in three specific Met–aromatic interactions (Fig. 5a). Here, the Met sulfur-aromatic interaction is of the order of 1–3 kcal/mol, as measured by an intermolecular distance of approximately 5.5 Å (d_Met–π_, the distance between sulfur and the ring center) and by an orientational preference of sulfur concerning the aromatic ring plane at around 30–60° (Ang_Met–π_, the angle between the sulfur position vector and the normal vector to the aromatic ring) (Fig. 5c).

With the refined NleB2 structure, we measured the distance d_Met–π_ between each ring center in F44, W46, and Y69 and the Met219 sulfur, as well as the angle Ang_Met–π_ between the normal vector to the aromatic ring and the Met219 sulfur position vector from each ring center; we thus obtained all active d_Met–π_ and Ang_Met–π_ scales within a distance of approximately 5.5 Å and nearing 50°, with concrete scales are [6.20 Å 5.35 Å 5.42 Å]/[5.99 Å 5.20 Å 5.44 Å] of d_Met–π_(F44, W46, Y69) and [48.44° 58.29° 66.52°]/[47.72° 52.25° 55.74°] of Ang_Met–π_(F44, W46, Y69) for each asymmetric chain (Fig. 5a,d).

### A single methionine mutation in NleB1 A222M Results in the loss of more than half of GlcNAcylation activity

**Figure 6:**
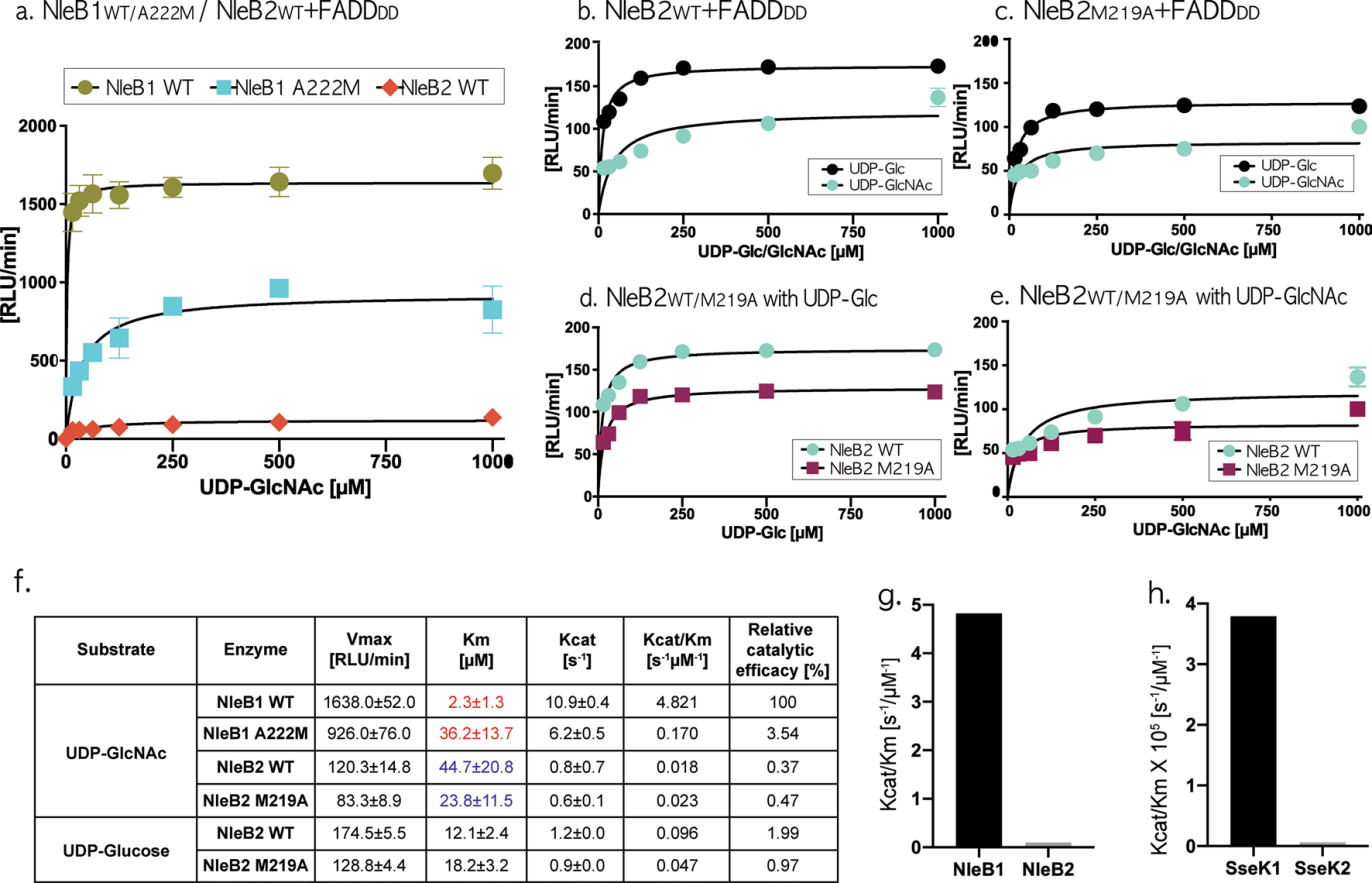
Kinetic analysis of NleB1/2 and their derivatives with FADD DD in the presence of UDP-Glc/GlcNAc. (**a**–**e**) Graphical representation of the Michaelis–Menten kinetics for NleB1, NleB2, and their variants, measured using a UDP-Glo assay. Each protein construct was purified by Ni-NTA and gel-filtration chromatography. The UDP-GloTM Glycosyltransferase assay (Promega) was performed as per the manufacturer’s instructions. The mean relative light units (RLU) detected from three replicates are shown with error bars representing the standard deviation. (**f**) Vmax, Km, and Kcat values are calculated using non-linear regression of the Michaelis–Menten equation in GraphPad Prism 8, shown with the standard deviation. (**g**,**h**) Bar chart showing the relatively weaker glycosyltransferase activity of NleB2 and SseK2 than that of NleB1 and SseK1, respectively; the data for SseK1/2 glycosyltransferase activity was obtained from Park et al.^2^.

Structural analysis suggested that, unlike the other NleB/SseKs DAD motif, the single methionine of NleB2 DMD, tightly tied in three Met–aromatic interactions, attenuates the propensity of aspartate to hold Mn^2+^ and the ligand, resulting in deficient glycosylation activity (Fig. 6a,g). Here, the wild-type NleB1 also has three aromatic F47, W49, and Y72 residues in the same positions as those of F44, W46, and Y69 in NleB2, possibly to form stable Met–aromatic interactions in the site-mutated NleB1 A222M (Fig. 5b). Upon replacing Met222 at Ala222 in the wild NleB1 structure, three Met–aromatic interactions for F47, W49, and Y72 residues were measured on the active scale within a distance of approximately 5.5 Å and an angle of 50°, such as [6.09 Å 5.15 Å 5.37 Å] of d_Met–π_(F47, W49, Y72) and [47.18° 57.55° 60.13°] of Ang_Met–π_(F47, W49, Y72) (Fig. 5b,d).

To examine whether replacing a single methionine between two aspartates in the DxD motif attenuates glycosylation activity, we obtained the NleB1 A222M variant by mutating Ala222 of wild NleB1 ^221^DAD^223^ to Met222 of NleB1 ^221^DMD^223^ and measured its UDP-GlcNAc binding affinity compared with that of the wild-type NleB1. The variant A222M showed a dramatically reduced reaction rate, exhibiting approximately 3.5% of the original activity in NleB1 (Fig. 6a,g), supporting that Met substitution between two aspartates of the catalytic DxD motif leads to a dramatic depletion of glycosylation activity. Surprisingly, the Km value of NleB1 A222M increased by approximately 18-fold, indicating a significant decrease in the UDP-GlcNAc binding affinity mediated by Mn^2+^ (Fig. 6f). Unlike the other NleB/SseKs effectors, the NleB2 preference for UDP-Glc over UDP-GlcNAc as a donor substrate was addressed following a recent study^33^ (Fig. 6b); further, the NleB2 M219A variant, which did not form three Met–aromatic interactions, also preferred UDP-Glc over UDP-GlcNAc, even less than that found with wild-type NleB2 (Fig. 6c). Here, the NleB2 M219A variant did not present a higher enzymatic activity in the presence of UDP-Glc/GlcNAc than that of the wild-type NleB2, unlike the dramatically reduced glycosylation in NleB1 A222M (Fig. 6d,e). However, the preference of NleB2 M219A for UDP-Glc over UDP-GlcNAc is more trivial than the wild NleB2 donor-selectivity (Fig. 6b–e) indicating that the introduction of an Ala to the DMD motif of NleB2 at least changes the glycosylation in the presence of UDP-GlcNAc compared to that in the other variants in terms of donor-selectivity and glycosylation. This could be explained by the less than 1% glycosylation efficiency in NleB2 compared to that of NleB1 and the weak sensitivity of NleB2 to the glycosyltransferase assay (Fig. 6f). However, the Km value of NleB2 M219A was reduced by approximately twofold compared to that of wild-type NleB2, indicating that the higher UDP-GlcNAc binding affinity mediated by Mn^2+^ after replacing Met with Ala and a single methionine substitution in the fatal NleB1 DAD motif was deficient in glycosylation activity reaching only 3.5% compared to that of the wild-type NleB1; thus, only one methionine in NleB1 A222M prevents catalytic aspartates from binding Mn^2+^ and the ligand and from GlcNAcylating the FADD death domain (Fig. 6f).

## Discussion

**Figure 7:**
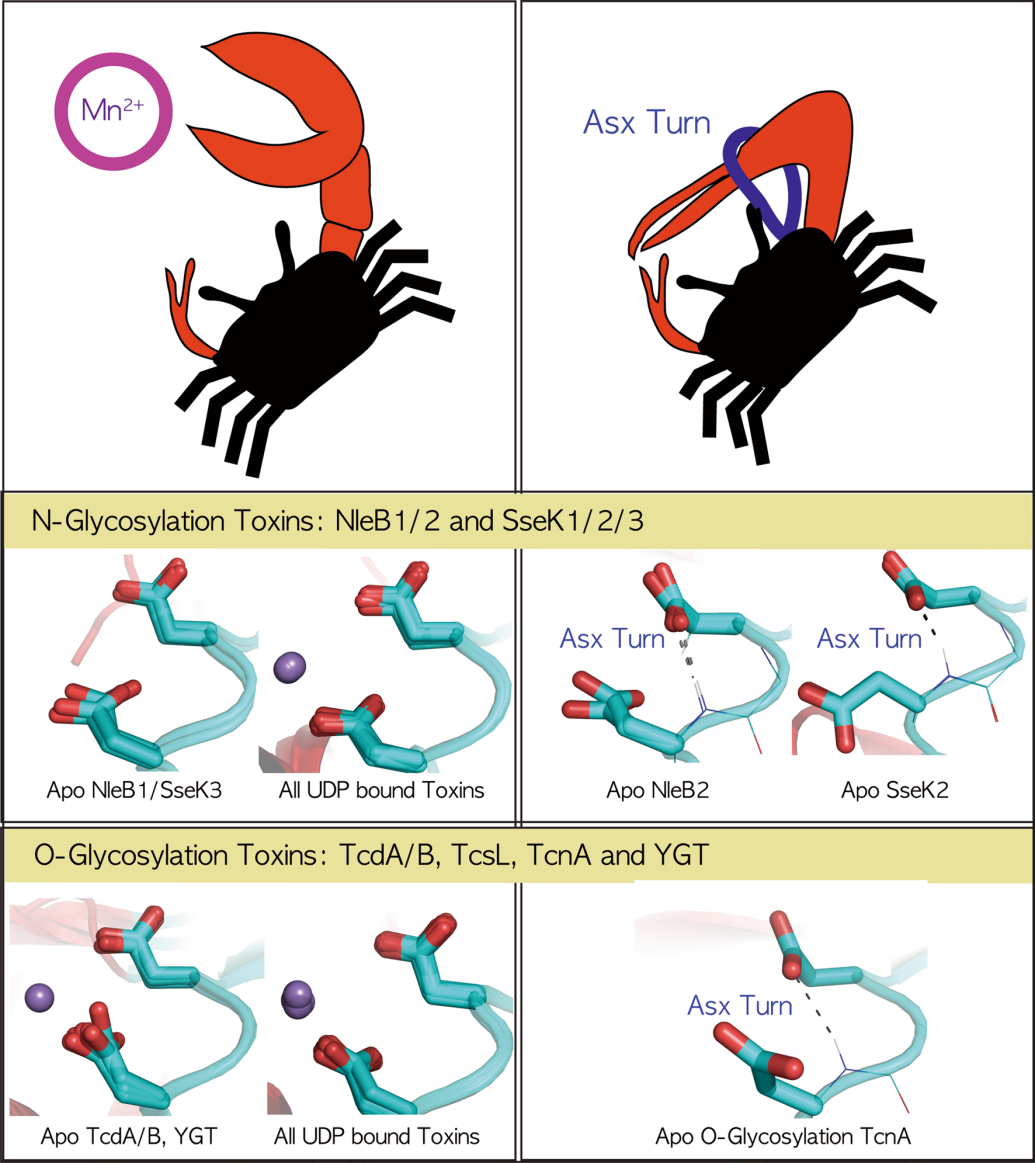
Cheliped-like Aspartate Dn of the DxD motif in bacterial glycosylation toxins. Except for the three toxins in apo NleB2, the SseK2 and TcnA toxins with a DxD motif tightly tied to the Asx-turn, the DxD motif in all glycosylation toxins is bent by the backbone H-bind in a Type-I-turn and permits the first catalytic aspartate of the DxD motif to freely catch Mn^2+^, as a crab-cheliped evolved into a pincher.

The DxD motif, which is conserved in all mono-glycosyltransferase bacterial toxins, is critical to tightly grasp the Mn^2+^ and sugar donor; mutation of this motif leads to deficient glycosylation. Surprisingly, the DxD motif in the apo large clostridial glycosylating cytotoxins is positioned similarly in the complex with the ligand, where the first aspartate weakly binds three-residues apart from tyrosine named as a YD bond, such that two catalytic oxygens of the aspartate sidechains can easily grasp Mn^2+^ and the donors. In the case of clostridial TcdA/B toxins, the nine asymmetric structures in all six known apo TcdA/B crystals indicate that DxD motifs (Asp285/Asp287 in TcdA and Asp286/Asp288 in TcdB) remain in the characteristic YD bond, capable of easily catching Mn^2+^ (Fig. 7); in particular, the DxD motif in two apo TcdA/B crystal structures (3SS1 and 5UQT) was crystalized with bound Mn^2+^, which proved that the characteristic conformation in the two aspartate sidechains of the TcdA/B DxD motif is suitable for Mn^2+^ binding (Fig. 4a-1/2). This also occurs in two NleB/SseKs orthologs: EPEC and EHEC NleB1, and *S. enterica* serovar Typhimurium SseK3 effectors. In the apo NleB1 and SseK3 structures, the two aspartate sidechains of DxD motifs (Asp226/Asp228 in SseK3 and Asp221/Asp223 in NleB1) maintain a suitable conformation to embrace Mn^2+^ and UDP-GlcNAc, similar to their UDP-bound structures (Fig. 7).

Unlike the fatally pathogenic NleB1 and SseK3 toxins, the *S. enterica* SseK2 effector with lower glycosylation activity (Fig. 6h) has a different conformation in the apo DxD motif from that in the ligand-bound state (Fig. 4c-5/d-5). In complex with UDP or UDP-GlcNAc ligands, it is still consistent with other NleB/SseK orthologs in that two Asp239 and Asp241 tightly bind Mn^2+^ and UDP/UDP-GlcNAc ligands (Fig. 4c-5). However, in the apo state, the oxygen Oδ in SseK2 Asp239 binds the amino group of Asp241 to form an Asx-turn instead of binding Oη of Tyr 237, to form the YD bond (Figs. 2c, 4c-5). This was also observed in the deficient EPEC and EHEC NleB2 in this study, where the Oδ of Asp218 formed an Asx-turn with the amino group of Asp220 rather than the YD bond with Oη Tyr216 (Figs. 2b and 4c-4). Thus, the specific Asx-turn breaks the YD bond in weak SseK2/NleB2 effectors, making it impossible for the catalytic aspartate to grasp its ligand without changing the side-chain conformation in the DxD motif (Figs. 2b,c, 7).

Here, NleB2 has one more handicap of a changed methionine between the two catalytic aspartates in the DMD (Asp-Met-Asp) motif, unlike that in the DAD (Asp-Ala-Asp) motif in the other NleB/SseKs orthologs. Although the reason remains unclear, the Met sulfur-aromatic interaction is considered to be active in the order of ~ 2 kcal/mol within an intermolecular distance of ~ 5.5 Å, (d_Met–π_, the distance between the sulfur and the ring center), and by a directional preference for sulfur concerning the aromatic ring plane around 30–60° (Ang_Met–π_, the angle between the sulfur position vector and the normal vector to the aromatic ring). Met–aromatic interactions are associated with several age-associated diseases, including Alzheimer's, Creutzfeldt–Jacob, and von Willebrand diseases, as well as NleB/SseK-relevant TNF ligand-receptor complexes, namely TRAIL-DR5 and LT-TNFR1. All of them have a single methionine within 5 Å of several aromatic residues, resulting in meaningful Met sulfur-aromatic interactions, wherein a single Met mutation results in worsened age-associated diseases or an overactive glycoprotein Ibα with a bleeding disorder. Similarly, in the deficient NleB2 effector, a single methionine in the NleB2 DMD motif is placed at the center of three aromatic residues (Phe, Tyr, and Trp) within 6.2 Å separation to form triple Met–aromatic interactions with the three neighboring aromatic rings at approximately 55°, inhibiting the DxD motif from a more suitable binding ligand than that in the NleB1 and SseK1/3 DAD motifs (Fig. 5a,c).

The rigid methionine in the NleB2 DMD motif interacts tightly with three aromatic residues at a 6 Å distance and near a 50° angle to attenuate NleB2 glycosylation (Fig. 6). A Met-mutation in the alanine of the wild NleB1 DAD motif results in tight Met–aromatic interactions in NleB1 A222M with three neighboring aromatic residues, resulting in decreased UDP-GlcNAc binding affinity and FADD-GlcNAcylation to 3.5% of that in the wild-type NleB2. Thus, a single methionine residue between two aspartate residues of the DMD motif in wild NleB2 and NleB1 A222M results in Met–aromatic interactions with the neighboring aromatic residues and attenuates Mn^2+^/UDP-GlcNAc binding affinity as well as FADD-glycosylation, unlike that of the other pathogenic NleB/SseKs DAD motifs.

Overall, this study suggests that it is necessary for all mono-glycosylation toxins, including NleB/SseKs orthologs, to actively N-GlcNAcylate their target proteins; the first aspartate sidechain in the catalytic DxD motif is free enough to grasp the cofactor Mn^2+^ and donor when its sidechain is not intertwined in the Asx-turn but when its backbone is tied in a Type-I-turn. This explains why the core aspartate sidechain of the DxD motif in SseK2/NleB2 effectors is intertwined in an Asx-turn and why a single methionine replacement in the NleB2 DxD motif caged in Met–aromatic bonds hinders the catalytic DxD motif from ligand binding, leading to more deficient SseK2/NleB2 GlcNAcylation than that with the other pathogenic NleB1/SseK1/SseK3 orthologs.

## Methods

### Site-directed mutagenesis of NleB1/2 genes

EHEC NleB1 (1–329) and NleB2 (1–326) genes were amplified by PCR and cloned into the pVFT3S vector (Korean patent 10-0690230), which has a 6xHis-thioredoxin (Trx) and tobacco tech virus (TEV) protease cleavage site. PCR-based site-directed mutagenesis was used to generate NleB1 A222M and NleB2 M219A. The FADD (1–192) gene was amplified by PCR from the synthesized DNA and cloned into a modified pET28a vector (Novagen), in which the thrombin cleavage site was replaced with the TEV protease site (Supp. Table 1).

### Protein expression and purification

All sub-cloned genes for FADD, Trx-NleB1, Trx-NleB2, and their variants were transformed into *E. coli* BL21 (DE3) (Novagen). Overnight LB cultures of each vector were used to inoculate high salt Luria-Broth medium. When the O.D_600_ reached 0.6–0.8, the temperature was decreased to 17 °C and the culture was induced with 0.1 mM IPTG (isopropyl 1-thio-β-d-galactopyranoside). After 16 h incubation, each protein was purified using nickel-affinity chromatography (Takara, #635662). The cell was lysed using lysis buffer of 20 mM Tris–HCl (pH 7.5), 300 mM NaCl, 30 mM imidazole, and the proteins were eluted using elution buffer of 300 mM imidazole in lysis buffer.

To improve protein purity, FADD was loaded into the gel-filtration chromatography (HiLoad 16/600 superdex 200 pg, Cytiva) carried out in SEC buffer of 50 mM Tris–HCl (pH7.5), 100 mM NaCl, 10 mM MgCl_2_, and 1 mM MnCl_2_ immediately after affinity chromatography.

For NleB1, NleB2, and their variants, the TEV protease cleavage site was cleaved using TEV protease. After desalting to binding buffer of 20 mM Tris–HCl (pH 7.5), 300 mM NaCl, 30 mM imidazole, 6XHis-TRX was removed using nickel-affinity chromatography. Each protein was further purified using gel filtration by the same protocol of FADD.

### Structure determination and refinement

Using the previously deposited NleB2 (1–326) structure (PDB ID:5H5Y), the hydrogen bond network in the DxD motif was resolved by newly orienting one water molecule and processing repeated refinements by molecular replacement using a UDP-bound SseK2 structure (5H63) with MOLREP^34^, REFMAC5^35^, and COOT^36^ for MR, refinement, and model building, respectively (Table 1). Structural figures are made using PyMOL. (PyMol Molecular Graphics Systems, Version 2.5.2; Schrodinger).

**Table 1:** Data collection and refinement statistics (molecular replacement).

	6AI4
**Data collection**	
Space group	P 21 2 21
Cell dimensions	
* a*, *b*, *c* (Å)	67.82, 95.52, 120.08
α, β, γ (°)	90, 90, 90
Resolution (Å)	2.10–67.8 (2.10–2.16)
*R*_sym_ or *R*_merge_	0.111 (0.941)
*I*/σ*I*	15.5 (3.3)
Completeness (%)	99.7 (99.9)
Redundancy	13.6 (14.0)
**Refinement**	
Resolution (Å)	2.10–44.42
No. reflections	43,743
*R*_work_/*R*_free_	26.05/29.8
No. atoms	4799
Protein	4662
Ligand/ion	0
Water	137
*B*-factors	49.54
Protein	49.73
Ligand/ion	–
Water	43.2
R.m.s. deviations	
Bond lengths (Å)	0.009
Bond angles (°)	1.23

### Structural analysis of the DxD motif in glycosyltransferase toxin

All 30 crystal structures of glycosyltransferase toxins were obtained from the RCSB PDB site, and a total of 51 chains in the asymmetric unit were separated into four groups including apo (11 chains) and ligand-bound O-glycosylation toxins (18 chains) and apo (7 chains) and ligand-bound N-glycosylation NleB/SseKs toxins (15 chains) in Table 2. Ten residues near the DxD motif (TcdA:281GVYL**DVD**MLP290/TcdB:282GMYL**DVD**MLP291/TcsL:282GMYL**DVD**MLP291/TcnA:280GVYC**DLD**FLP289/YGT:207GVYI**DTD**LLP216/SseK1:219CIYL**DAD**MII228/SseK2:235CIYL**DAD**Mll244/SseK3:222CIYL**DAD**Mll231/NleB1:217CIYL**DAD**MII226/NleB2:214CIYL**DMD**MIL223) was structurally aligned to each ten-residue region of TcdA (3SS1 and 3SRZ), SseK3 (6EYR, A chain), and SseK2 (5H63, A chain), with respect to O-/N-linkage and ligand-binding.

**Table 2:** Bacterial mono-glycosyltransferase toxins.

Mono-glycosylation bacterial toxins	PDB ID (chain)	Resolution (Å)	Ligand	References
**O-linked toxin**				
Ligand free toxin				
TcdA	3SS1	2.2	Apo (Mn)	^38^
	4DMV	1.5	Apo	^39^
TcdB	5VQM(A)	2.79	Apo	^40^
	5VQM(B)			
	5UQT(A)	2.75	Apo (Mn)	^41^
	5UQT(B)			
	6OQ8(A)	2.2	apo	^3^
	6OQ8(B)			
**TcnA**	**2VK9**	**2.85**	**Apo**	^5^
YGT	6RTH(A)	3.5	Apo	^6^
	6RTH(B)			
Ligand bound toxin				
TcdA	4DMW	2.5	UDP + Mn	^39^
	3SRZ	2.58	UDP-Glc + Mn	^38^
	5UQK	1.85	U2F + Mn	^41^
	5UQL	1.97	U2F + Mn	
TcdB	6OQ7	2.39	UDP-Glc + Mn	^3^
	2BVL	2.2	UDP-Glc + Mn	^42^
	2BVM	2.55	UDP-Glc + Mn	
	5UQM	2.03	U2F + Mn	^41^
	5UQN	2.06	U2F + Mn	
TcsL	2VKD(A)	2.53	UDP-Glc + Mn	^5^
	2VKD(B)			
	2VKD(C)			
	2VKH(A)	2.3	UDP-Glc + Mn	
	2VKH(B)			
	2VKH(C)			
	2VK8(A)	2.31	UDP + Ca	
	2VK8(B)			
	2VK8(C)			
YGT	6RTG	1.9	UDP + Mn	^6^
**N-linked toxin**				
Ligand free toxin				
NleB1	6E66	2.1	Apo	^15^
**NleB2**	**6AI4(A)**	**2.1**	**Apo**	**This study**
	**6AI4(B)**			
SseK3	6EYR(A)	2.2	Apo	^16^
	6EYR(B)			
**SseK2**	**5H61(A)**	**1.86**	**Apo**	^2^
	**5H61(B)**			
Ligand bound toxin				
NleB1	6ACI	1.87	UDP + Mn + FADD	^15^
SseK2	5H62(A) 5H62(A)	1.66	UDP + Mn	^2^
	5H62(B)			
	5H63(A)	1.92	UDP-GlcNAc + Mn	
	5H63(B)			
	5H63(C)			
	5H63(D)			
SseK3	6EYT(A)	2.21	UDP + GlcNAc + Mn	^16^
	6EYT(B)			
	6CGI(A)	2.3	UDP	^17^
	6CGI(B)			
	6CGI(C)			
	6CGI(D)			
	6DUS(A)	2.6	UDP + GlcNAc + Mg	
	6DUS(B)			

All 30 crystal structures of glycosyltransferase toxins were obtained from the RCSB PDB site, and a total of 51 chains in the asymmetric unit were separated into four groups consisting of apo (11 chains) and ligand-bound O-linked toxins (18 chains) and apo (7 chains) and ligand-bound N-linked NleB/SseK toxins (15 chains). Bold toxins in TcnA, NleB2, and SseK2 have an Asx-turn in the catalytic DxD motif, which is tightly tied to the first aspartate side chain of the DxD motif. The other toxins have a free side chain of the first aspartate in the DxD motif only tied in the backbone C10 H-bond Type-I-turn, one of the β turns.

### Glycosylation reaction

The kinetic parameters Vmax and Km were calculated using UDP-Glo assays (Promega, #6972), similar to those in previous studies^33,37^. Purified NleB1, NleB2, and their variants were diluted to 150 nM and incubated with a mixture of substrates, 1 μM FADD in 1X glycosyltransferase buffer (50 mM Tris–HCl pH 7.5, 100 mM NaCl, 10 mM MgCl_2_, and 1 mM MnCl_2_) with titrated UDP-GlcNAc (series of 8 dilutions, 0–1 mM) for 30 min at 30 °C. The UDP-Glo assay was then performed according to the manufacturer's instructions, and luminescence was detected using a CLARIO star microplate reader (BMG Labtech). The relative light units (RLU) measured at the end of the reaction were divided by 30 to calculate the RLU/min unit in order to depict the dependence of catalytic activity on the UPD-GlcNAc concentration. Vmax and Km values were obtained using the non-linear regression fitting of Michaelis–Menten equation in GraphPad Prism version 8.4.0.

### Measure Met–aromatic interaction

All Met–aromatic interactions near the DxD motif were extracted by the definition^32^, in which Met–aromatic energy is associated with both the distance d_Met–π_ between the sulfur of Met and the ring center C_π_ of three aromatic residues, Phe, Tyr, and Trp, and the orientation angle Ang_Met–π_ between the positional vector of sulfur from the ring center and the normal vector n_π*,*_ defined by the plane of the aromatic ring, as follows:

- C_π_ ≡ coordinate of ring center in Phe, Tyr, and Trp residues;
- C_π_ (Phe or Tyr) ≡ {sum of three coordinates: CG, CE1, and CE2 atoms}/3
- C_π_ (Trp) ≡ {Sum of three coordinates CD2, CZ2, and CZ3 atoms]/3
- n_π_ ≡ the normal vector defined by the plane of the aromatic ring;
- n_π_ (Phe or Tyr) ≡ cross product [{CE1-CG}, {CE2-CG}].
- n_π_ (Trp) ≡ cross product [{CZ2-CD2}, {CZ3-CD2}].
- S ≡ sulfur coordinate of Met;
- d_Met–π_ ≡ Distance (S, C_π_);
- Ang_Met–π_ ≡ Angle [{S-C_π_}, n_π_].

The scales of Met–aromatic energy are calculated by MatLab program with all atom coordinates of the Met and aromatic Phe, Tyr, and Trp residues in their PDB files.

## Supplementary Information

Supplementary Table 1.

## Data Availability

The collected data in this study are available from the corresponding author on reasonable request.
