# Author Correction: Catalytic DxD motif caged in Asx-turn and Met–aromatic interaction attenuates the pathogenic glycosylation of SseK2/NleB2 effectors

**DOI:** 10.1038/s41598-023-27966-w

**Published:** 2023-01-17

**Authors:** Eunhee Koh, Uijin Kim, Hyun-Soo Cho

**Affiliations:** Department of Systems Biology, College of Life Science and Biotechnology, Yonsei University, Seoul, 03722 Republic of Korea

Correction to: *Scientific Reports* 10.1038/s41598-022-22803-y, published online 11 November 2022

The Acknowledgements section in the original version of this Article was incomplete.

“This work was supported by the National Research Foundation of Korea (NRF) grant funded by the Ministry of Science and ICT (2021R1A2C3010506, 2016R1A5A1010764, 2017M3A9F602975, 2019M3E5D6063903, 2019K1A3A1A4700071).

now reads:

“This work was supported by the National Research Foundation of Korea (NRF) grant funded by the Ministry of Science and ICT (2021R1A2C3010506, 2016R1A5A1010764, 2017M3A9F602975, 2019M3E5D6063903, 2019K1A3A1A4700071, 2021M3A9I2080491).”

The original Article has been corrected.

