# Supplementary material for: Catalytic DxD motif caged in Asx-turn and Met–aromatic interaction attenuates the pathogenic glycosylation of SseK2/NleB2 effectors
Source: Sci Rep. 2022 Nov 11;12:19288. doi: 10.1038/s41598-022-22803-y (PMC9652389; doi:10.1038/s41598-022-22803-y)
Supplement: Supplementary file 1 — Supplementary Table 1. [file 41598_2022_22803_MOESM1_ESM.doc]

Supplementary Table1. Primers used in this study

| **description (primer)** | **sequence** |
| --- | --- |
| NleB1 A222M F | ATATATCTTGATATGGACATGATTATT |
| NleB1 A222M R | AATAATCATGTCCATATCAAGATATAT |
| NleB2 M219A F | ATCTATCTTGATGCGGATATGATACTT |
| NleB2 M219A R | AAGTATCATATCCGCATCAAGATAGAT |
